# Identification and Characterization of a Bacterial Homolog of Chloride Intracellular Channel (CLIC) Protein

**DOI:** 10.1038/s41598-017-08742-z

**Published:** 2017-08-17

**Authors:** Shubha Gururaja Rao, Devasena Ponnalagu, Sowmya Sukur, Harkewal Singh, Shridhar Sanghvi, Yixiao Mei, Ding J. Jin, Harpreet Singh

**Affiliations:** 1 Department of Pharmacology and Physiology, Drexel University College of Medicine, Philadelphia, PA 19102 USA; 2 Protein Biochemistry, Life Sciences Research, Beckman Coulter Inc, 11800 SW 147th Ave, Miami, FL 33196 USA; 3 RNA Biology Laboratory, National Cancer Institute-Frederick, NIH, MD 21702 USA; 4 Division of Cardiology, Department of Medicine, Drexel University College of Medicine, Philadelphia, PA 19102 USA

## Abstract

Chloride intracellular channels (CLIC) are non-classical ion channels lacking a signal sequence for membrane targeting. In eukaryotes, they are implicated in cell volume regulation, acidification, and cell cycle. CLICs resemble the omega class of Glutathione S-transferases (GST), yet differ from them in their ability to form ion channels. They are ubiquitously found in eukaryotes but no prokaryotic homolog has been characterized. We found that indanyloxyacetic acid-94 (IAA-94), a blocker of CLICs, delays the growth of *Escherichia coli*. *In silico* analysis showed that the *E. coli* stringent starvation protein A (SspA) shares sequence and structural homology with CLICs. Similar to CLICs, SspA lacks a signal sequence but contains an omega GST fold. Electrophysiological analysis revealed that SspA auto-inserts into lipid bilayers and forms IAA-94-sensitive ion channels. Substituting the ubiquitously conserved residue leucine 29 to alanine in the pore-forming region increased its single-channel conductance. SspA is essential for cell survival during acid-induced stress, and we found that acidic pH increases the open probability of SspA. Further, IAA-94 delayed the growth of wild-type but not *sspA* null mutant *E. coli*. Our results for the first time show that CLIC-like proteins exist in bacteria in the form of SspA, forming functional ion channels.

## Introduction

The chloride intracellular channel (CLIC) proteins are well conserved in most eukaryotic species^1^. The founding member of CLICs, p64 was purified from bovine kidney extracts through its affinity to a chloride current blocker, indanyloxy acetic acid 94 (IAA-94)^2^. They are a unique class of ion channels with a property to exist as both soluble and membrane forms^1, 3–5^. Crystal structures of soluble CLICs^6–11^ show structural homology to the glutathione S-transferases (GST)^12^ superfamily but differ from them as they can auto insert into membranes to form functional ion channels^1^. CLIC proteins defy traditional ion channel biology by lacking a signal sequence and yet insert into membranes to form channels^4^. Non-canonical properties of CLICs have placed them along with metamorphic proteins^13^ such as annexins and bacterial toxins^14, 15^. CLIC family consists of six mammalian paralogs (CLIC1-6)^1, 4^, one *Drosophila melanogaster* (*Dm* CLIC)^8^, two *Caenorhabditis elegans* (*Ce*, EXC4, and EXL1)^16^ and four *Arabidopsis thaliana* proteins (*At* DHAR 1–4)^17^.

Emerging data over the last two decades from large-scale genome analysis as well as electrophysiology have indicated the presence of several ion channels in bacteria and archaea. Although several classes of CLIC-like proteins such as stringent starvation protein A (SspA) are predicted to exist in prokaryotes^3, 18^, no homologs were characterized so far. In this study, we focused on studying structural similarities and establishing ion channel activity of a CLIC-related prokaryote member, SspA, found on the basis of *in silico* analysis^18^. SspA is a ~24.5 kDa protein, well conserved among Gram-negative bacteria, and its homologs are present in human, animal, plant and insect pathogens^19^. It is predominantly synthesized under conditions of extreme amino acid starvation^18, 20–24^. SspA is a RNA polymerase-associated protein required for transcriptional activation of bacteriophage P1 late genes^25^. *sspA* mutants show impaired transition from early to late gene expression during P1 bacteriophage lytic cycle^26^ due to its activity as a transcriptional activator^25^. *sspA* mutants are also less viable during starvation or stationary phases in accordance with its predicted role to regulate gene expression under nutrient starvation^27^.

Crystal structure of *Yersinia pestis* (*Yp*) SspA revealed a characteristic fold of GST^19^. However, the protein lacked GST activity and did not bind to glutathione^23, 28^. The structure revealed three distinct flexible regions of SspA, amino (N)- and carboxyl (C)-termini, and the alpha helix-2. Similar to SspAs, CLICs also have flexible luminal N- and a highly conserved ~200 residues C-terminus domain. The N-terminus of CLICs is predicted to unfold and refold to form functional ion channels in planar lipid bilayers^1^. However, no study has tested if SspA can also insert into the membranes to form functional ion channels.

Even though the physiological roles of higher organism CLIC proteins are being established, characterizing a prokaryotic homolog is important. The information gained from bacterial proteins could be extrapolated to study mammalian channels given the diversity of bacteria, and adaptability to extreme environmental conditions such as salinity, toxicity or acidity that allow them to survive^29^. We discovered that a CLIC blocker, IAA-94, delays the growth of *Escherichia coli* (*E. coli*). To characterize the prokaryotic homolog of CLICs, we have compared sequences and crystal structures of SspA^23^ with CLICs^6, 8, 11^. Here, we provide evidence for the first time that recombinant SspA reconstituted in planar lipid bilayers exhibits ion channel activity. We further explore biophysical properties of SspA by structure-function studies and show that similar to CLICs, SspA channel currents are blocked by a known CLIC protein blocker, IAA-94, reinforcing the idea that SspA proteins are related to CLICs. In addition, we have identified a ubiquitously conserved leucine residue that modulates the properties of SspA channels. We also found that the lower pH increases the open probability of the channel. Finally, using *sspA* mutants in *E. coli*, we have shown that IAA-94 delays the growth of wild-type but not the null mutant bacteria. Taken together, we have characterized the bacterial CLIC homolog, SspA, determined its biophysical properties as an ion channel, shown that it forms an acid-sensitive channel and its expression is essential for IAA-94 mediated reduction in growth.

## Results

### *E. coli* growth slowed down by IAA-94

**Figure 1:**
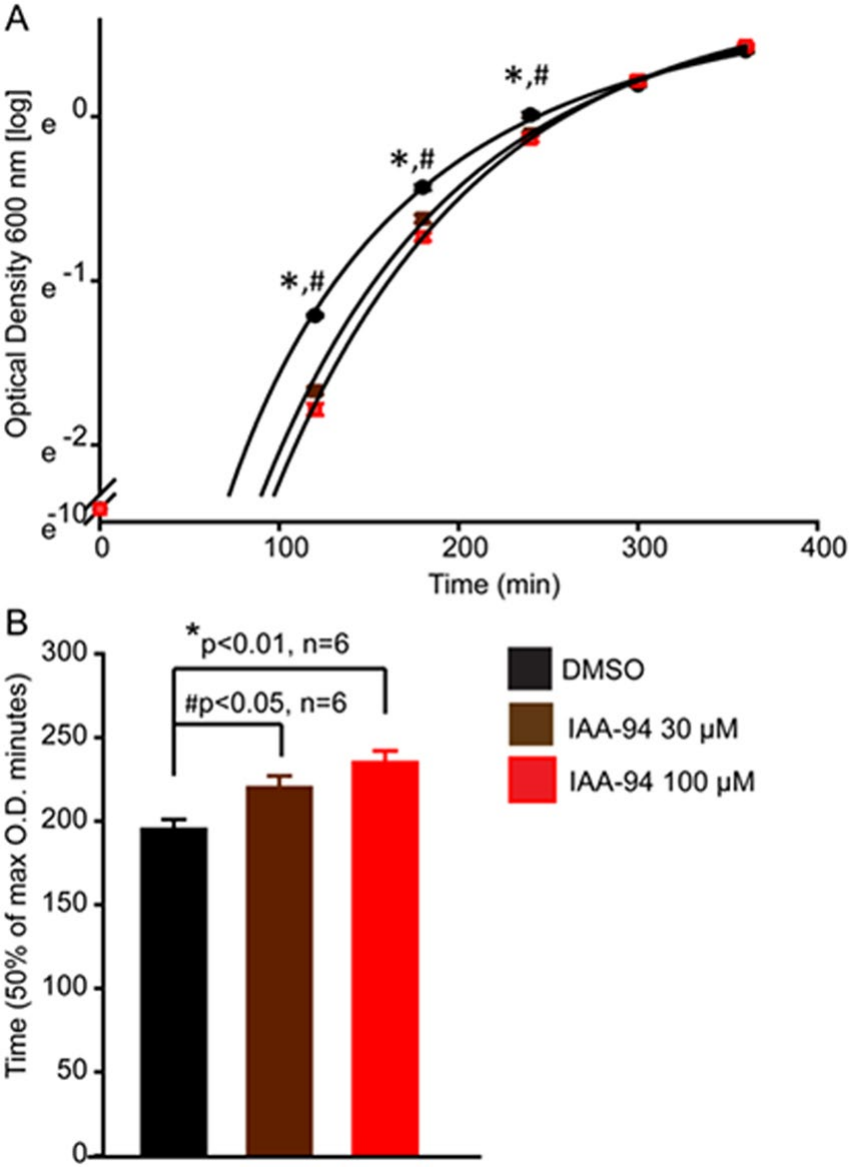
IAA-94 delays growth of *Escherichia coli*. (**A**) Growth curves of *E. coli* at 37 °C in the presence of IAA-94 (30 and 100 µM) and DMSO (control). Optical density (O.D) was measured at 600 nm every 60 minutes for six independent experiments and a log of O.D. was plotted *vs*. time. Data was fitted with Weibull distribution using Sigma plot. ‘*’ represent a significant difference from DMSO (blue). (**B**) Bar graphs representing a comparison of time to reach 50% growth. Significant differences were observed at 30 µM and 100 µM concentrations as compared to the DMSO control. Error bars in A and B represent standard error of the mean.

IAA-94 is a known blocker of CLICs and at lower concentrations (≤100 µM), it is specific for CLIC proteins^1^. In order to screen for CLIC-like channels in *E.coli*, we tested whether IAA-94 could affect the growth of *E. coli*. The addition of IAA-94 in the culture medium delayed the growth of *E. coli* at 37 °C, which was directly dependent on the dose of IAA-94 (Fig. 1A). We measured the time taken by *E. coli* to reach 50% growth and found that 30 µM and 100 µM IAA-94 significantly delayed the growth (Fig. 1A and B). At 30 µM and 100 µM, 50% growth was achieved at 220 ± 8 min, and 226 ± 12 min, respectively as opposed to 198 ± 4 min in DMSO control. Susceptibility towards IAA-94 was consistently observed during early log phase but not in stationary phase given bacteria become more resistant to environmental stress during slowing down of the growth^30^. The suppression of *E. coli* growth in the presence of IAA-94 implies that IAA-94-sensitive proteins are present in prokaryotes.

**Figure 2:**
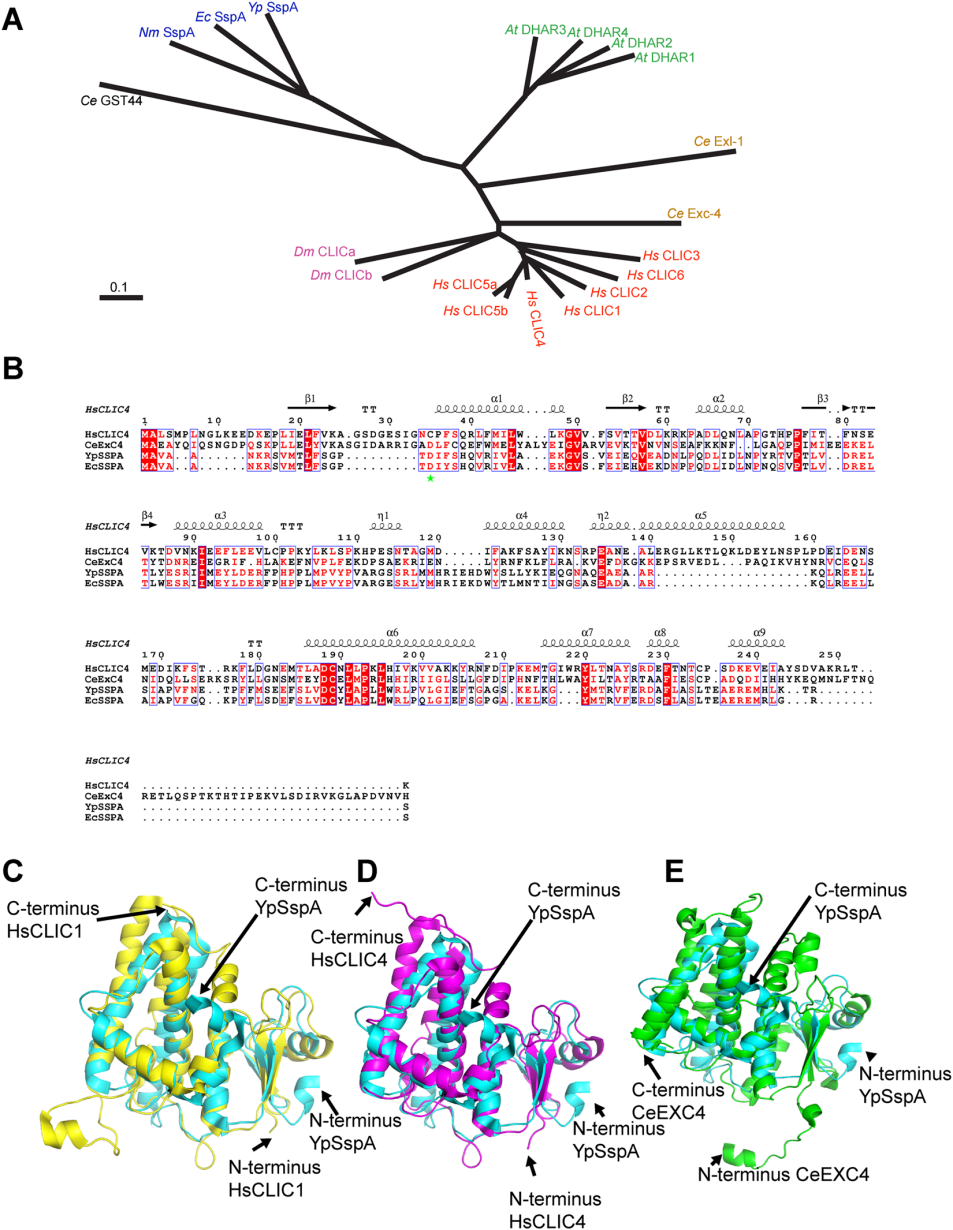
A prokaryotic homolog of CLIC proteins. (**A**) Phylogenetic tree of mammalian CLICs (red), *Dm* CLICs (pink), *Ce* CLICs (brown), *At* CLICs (green), bacterial CLICs (blue) and GST from *Ce* (black) showing CLICs diverged from bacterial counterparts. **(B)** Sequence alignment of *Yp* SspA, *Ec* SspA, *Hs* CLIC4 and *Ce* EXC4. Boxed regions indicate putative transmembrane domains (TMD1 and TMD2, respectively), red, α helices and yellow, β strands. Conserved amino acids are marked by ‘*’, ‘:’, and ‘.’ symbols for residues conserved in four, three and two sequences, respectively. The total number of amino acids in *Yp* SspA, *Ec* SspA, *Hs* CLIC4 and *Ce* EXC4 and are 213, 212, 253, and 290, respectively. Superposition of **(C)** *Yp* SspA (PDB code 1YYL, blue) and *Hs* CLIC1 (1KOM, yellow), (**D**) *Yp* SspA (blue) and *Hs* CLIC4 (PDB code 2AHE, magenta), (**E**) *Yp* SspA (blue) and *Ce* CLIC homolog EXC4 (PDB code 2YV9, green). All figures were prepared with PyMOL4. Extra helices in *Ce* are marked with arrows. The position of N- and C-termini are also highlighted.

To find IAA-94 sensitive proteins in bacteria we performed CLUSTAL alignment and CORAL analysis^31^. We found SspAs as conserved homologs of CLICs in several species of Gram-negative bacteria (Fig. 2A). The bacterial SspAs share homology with omega GSTs as shown in Fig. 2A and described earlier by Oakley^18^, but reported to lack GST-like activity^23^. Sequence alignment of two SspA sequences from *Yp* and *Ec* with *Hs* and *Ce* CLICs showed that these proteins have several conserved regions^3, 6, 8, 23^ (Fig. 2B). They have 12 α helices and 2 β sheets lining at similar positions as in mammalian or nematode CLICs (Fig. 2B). The cysteine (C) 24 of CLIC1 (C35 in CLIC4), involved in redox regulation of CLICs^32^ is substituted by an aspartate (D) in *Yp* SspA, *Ec* SspA, and *Ce* EXC4 (highlighted by green asterisk), however, the only cysteine in the C-terminus (α-helix 6) of *Yp* SspA is also conserved in most of the eukaryotic CLICs (Fig. 2B). There are several conserved leucines in the secondary structure of proteins including leucine 29 in the predicted transmembrane domain^32^. Supplementary Table 1 summarizes the sequence homology scores for mammalian, *Dm, At, Ce* CLICs with *Yp* SspA, *Nm* SspA, *Ec* SspA and *Ce* GST-44. *Yp* SspAs are closer to *At* DHAR1-3 and amongst mammalian CLICs to *Hs* CLIC4 (Supplementary Table 1).

We analyzed the known structures of *Yp* SspA^23^ in comparison to CLICs and GST, using SSM (PDB eFold), DALI and CE servers. Numbers of residues aligned (Nalign), Z scores and percentage sequence identities are listed in Table 1. Based on the scores in Table 1, we found that *Yp* SspA shares structural similarity with *Hs* GST, *Hs* CLIC4, *Hs* CLIC1 and *Ce* CLIC homolog EXC4. We further analyzed the structures manually (Fig. 2C,D,E) and found a two-domain GST-like architecture. Upon evaluation, it seemed that despite low sequence identity, overall topology of CLIC and SspA structures is similar. Intriguingly, *Hs* CLIC4, *Hs* CLIC1, and *Ce* CLIC homolog EXC4 appear to have longer N- and C-termini as compared to SspA. In summary, we corroborate^11, 18^ that SspA structure folds similar to *Hs* CLIC1, *Hs* CLIC4, and *Ce* CLIC homolog EXC4, which prompted us to investigate if SspA indeed forms functional ion channels similar to CLICs.

**Table 1:** Comparison of SspA with GTS, CLIC1, CLIC4 and EXC-4.

Comparison of alignments from SSM, DALI and CE			
PDB 1 PDB 2	SSM (N_align_/RMSD) Zscore (%si)	DALI (N_align_/RMSD) Zscore (%si)	CE (N_align_/RMSD) Zscore (%si)
1YY7(SspA) 1EEM (HsGST)	(189/2.47) 10.4(24)	(199/2.60) 21.8 (24)	(200/2.60) 6.92(22.5)
IYY7 (SspA) 2AHE (HsCLIC4)	(171/2.36) 8.9(23)	(191/3.1) 17.1(21)	(184/2.86) 6.23(18.5)
1YY7(SspA) 1K0M (HsCLIC1)	(165/2.15) 9.7(21)	(187/2.7) 17.0(19)	(186/2.72) 5.99(16.2)
1YY7(SspA) 2YV9 (CeCLIC homologue EXC-4	(172/2.55) 7.3(17)	(187/3.1) 14.8(18)	(179/2.62) 5.99(12.6)

%si = % sequence identity by individual program. MSD = root mean square distance between PDB1 and PDB2. Comparison of crystal structures of CLICs with SspA. Comparison of alignments from SSM, DALI, and CE for CLICs, and SspA.

### Formation of SspA ion channels in planar lipid bilayers

**Figure 3:**
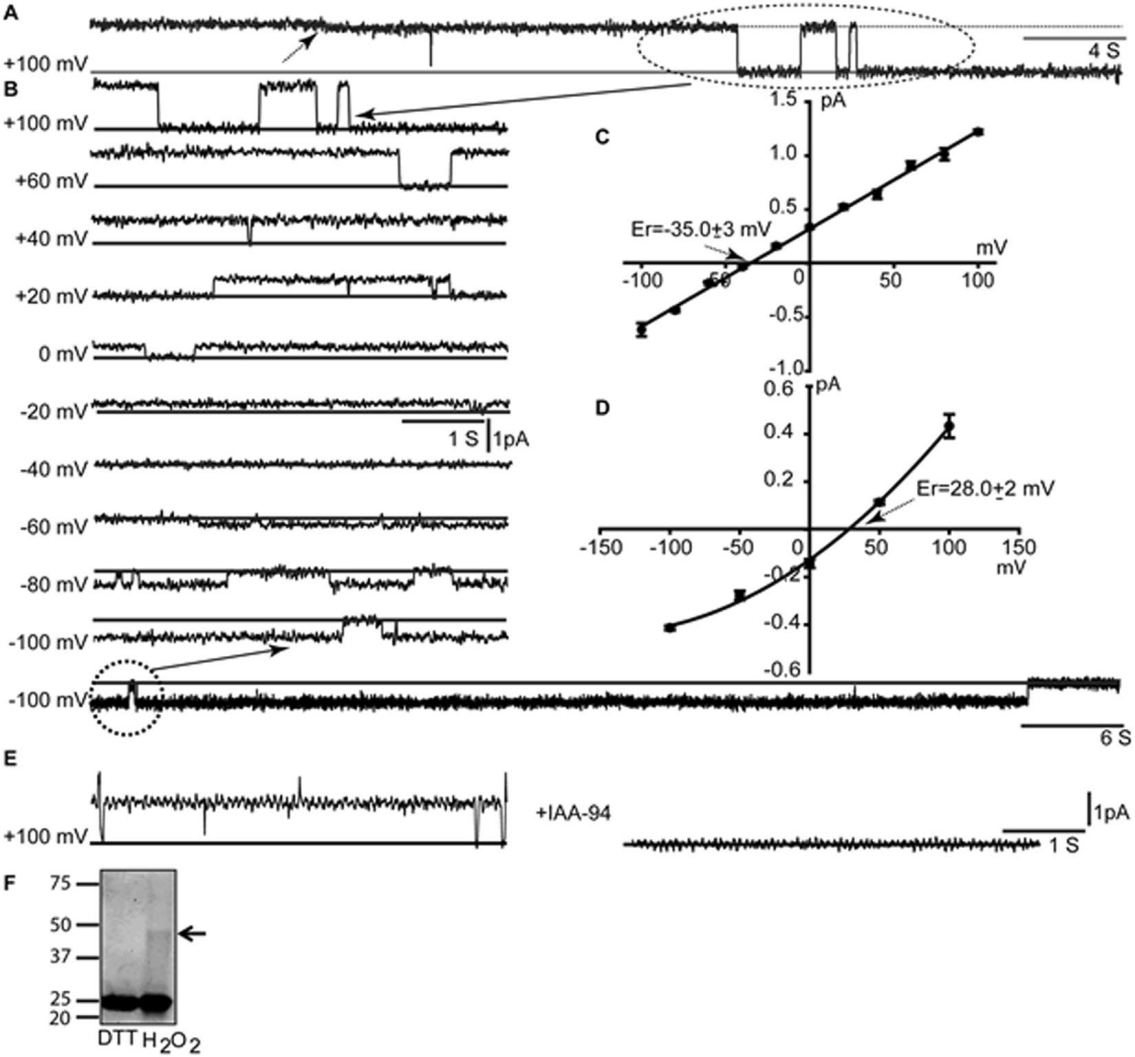
Single-channel properties of *Ec* SspA. Purified recombinant proteins were reconstituted in planar lipid bilayers at 500 mM:50 mM::cis:trans-KCl. (**A**) Representative trace of SspA at + 100 mV. A sub-state is indicated by a small arrow in the trace at the top. (**B**) Traces of single-channel currents of SspA at positive and negative holding potentials. The solid line indicates the closed level. (**C**) Current vs voltage (I/V) curve of SspA in asymmetric KCl. The single-channel conductance was 9.0 ± 1.5 pS (n = 8). The reversal potential was −35.0 ± 3.0 mV. (**C**) Current vs voltage (I/V) curve of SspA in 500 mM:50 mM::cis:trans TrisCl. Error bars represent standard error of the mean. (**E)** Representative traces showing that IAA-94 (100 µM, n = 5) blocked the SspA channel activity. Reversal potential was 28.0 ± 2.0 mV, indicating an anion-selective channel. Error bars represent standard error of the mean. **(F)** SDS-PAGE (10%) polyacrylamide gel of SspA in the presence of H_2_O_2_ (cropped from full-length gel shown in supplementary figure 3F). The arrow indicates the position of a dimer.

In order to investigate whether SspA can form functional ion channels, we employed planar lipid bilayers where CLIC proteins have been shown to form ion channels^32–34^. We added 1-palmitoyl-2-oleoyl phosphatidylethanolamine (POPE), PO-phosphatidylserine (POPS), and cholesterol in a molar ratio of 4:1:1 in 1 mM DTT previously reported suitable for obtaining consistent CLIC activities^32^. We reconstituted recombinant *Ec* SspA, a protein highly similar (83.49%) to *Yp* SspA^23^, in bilayers made of the same lipid mixture and obtained ion channel activity in 35 out of 50 independent experiments in the presence of 1 mM DTT (Fig. 3A–C). Single-channel current analysis revealed a conductance of 9.0 ± 1.5 pS (n = 8) (Fig. 3C). There was at least one sub-conductance level detected (Fig. 3A) at positive holding potentials. The channels showed long open durations at positive as well as negative holding potentials. The reversal potential (E_r_) of both substrates was found to be −35.0 ± 3.0 mV, implying that the channel preferably allows K^+^ ions over Cl^−^ ions (P_K_/P_Cl_ was 6.2 ± 1.2, n = 3) (Fig. 3C) in planar lipid bilayers. We also reconstituted channels in 500 mM TrisCl *cis vs*. 50 mM TrisCl *trans* (pH 7.4 and 1 mM DTT) and noticed the formation of channels within 15 min of addition of proteins. However, single-channel conductance was smaller (5.5 ± 0.5 pS, n = 3, Fig. 3D) as compared to the channels reconstituted in KCl gradient. The E_r_ was found to be 28.0 ± 2.0 mV, n = 3, for the SspA mediated currents in TrisCl gradient solutions (Fig. 3D). A dramatic change of E_r_ indicates improved selectivity for anions under TrisCl, and the channels were more permeable to Cl^−^ ions over K^+^ ions (P_Cl_/P_K_ was 4.1 ± 0.4, n = 3). We have also tested inter anionic selectivities for I^−^ and Br^−^ in comparison with Cl^−^. We found that under symmetrical salt activities, the anion permeability calculated from the equilibrium potential was I^−^ (1.3 ± 0.1) ≥Br^−^ (1.1 ± 0.2) ≥Cl^−^ (set to 1.0) (all mean ± SEM, n = 4–5 independent experiments). These results were comparable to mammalian CLICs^32^, and anion selective channels reported earlier in dog tracheal epithelium^35^.

As stated earlier, IAA-94 was originally used to purify CLIC proteins from kidney^2^, and it is extensively used to block CLIC-mediated currents^36–38^. Since SspA channels formed larger channels under KCl, we tested the effect of IAA-94 on channels reconstituted in KCl (Fig. 3E). Single-channel currents were recorded for 60 seconds before and after the addition of IAA-94. Addition of 100 μM IAA-94 decreased the open probability of SspA-mediated channel currents from 0.6 ± 0.25, n = 3 to 0.1 ± 0.05, n = 5. Near complete blockage of SspA-mediated currents provides pharmacological evidence that indeed SspA channel currents are similar to IAA-94-sensitive CLIC currents.

Mammalian CLIC proteins are known to form single channels in the presence of DTT as well as H_2_O_2_. We also attempted (n = 10) to reconstitute SspA in the presence of H_2_O_2_ but no channel activity was observed after 60 mins of addition of proteins in planar lipid bilayers. Recombinant proteins were also incubated with DTT or H_2_O_2_ which resulted in the appearance of small amounts of dimers of SspA in the presence of H_2_O_2_ (Fig. 3F) as shown by a band at ~50 kDa in addition to the predominant monomeric form (~25 kDa). Dimerization could possibly prevent the insertion of proteins into the bilayer under oxidizing conditions, and partially explains the absence of channel activity in presence of H_2_O_2_.

### Leucine 29 alanine (L29A) mutation changes biophysical properties of SspA

**Figure 4:**
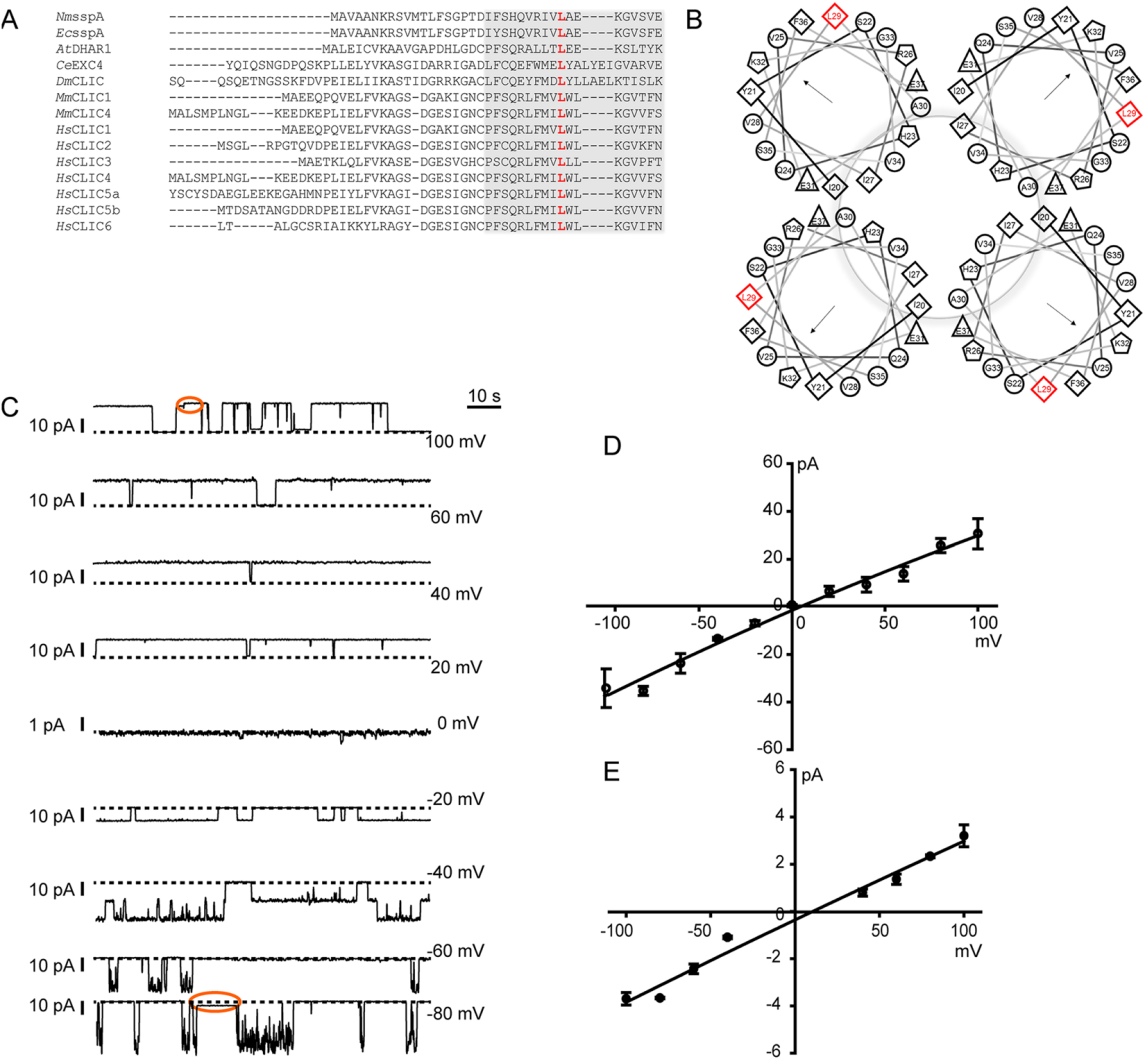
SspA L29A mutants increase the channel conductance. (**A**) Sequence alignment of the N-terminus of prokaryotes (*Nm* and *Ec*). Plants (*At*), worms (*Ce*), flies (*Dm*), mouse (*Mm*) and Humans (*Hs*) CLICs. Grey area represents the transmembrane domain in the N-terminus. The conserved leucine is represented in red. (**B**) Helical wheel projection of the putative transmembrane domain of *Ec*SspA. The L29 residue (red diamond) faces the lipid bilayer. Arrows indicate hydrophobic region. (**C**) Representative traces of SspA L29A channel currents at various holding potentials. The dashed line indicates closed levels. Substate levels are indicated by red ovals. (**D**) Current-voltage (I/V) plot of the large channel currents. (**E**) I/V plot of the substate level channel currents of SspA L29A. Reversal potential was found to be ∼ −3 ± 2 mV for both I/Vs. Error bars represent standard error of the mean.

CLIC proteins have a single transmembrane domain located in the N-terminus region which inserts into the bilayer to form a functional channel^1^. On sequence analysis (Figs 2B and 4A), the L29 was the only residue found to be conserved in all species studied, from bacteria to humans as well as amongst other mammalian CLIC homologs (CLIC1-6). On a closer analysis, the L29 in SspA was found to be strategically located facing the protein-lipid interface in the transmembrane domain shown to form a channel pore in mammalian CLICs (Fig. 4A,B). To understand the role of L29, the residue was mutated to alanine (A). Recombinant SspA protein containing L29A mutation dramatically increased the current amplitude of SspA channels (Fig. 4C) in planar lipid bilayers. The single channel conductance (Fig. 4D) of SspA L29A mutant was found to be 320 ± 30 pS (n = 10). A prominent substate was observed at all holding potential and the single channel conductance of the substate was 31 ± 5 pS (n = 10) (Fig. 4E). The susbtate was always associated with the larger channel openings (n = 10). Even though the single channel conductance was augmented due to L29A, the channel remained non-selective similar to the wild-type channels in planar lipid bilayers. Open probabilities of SspA (Fig. 3B) and SspA L29A (Fig. 4C) were found to be similar (Po = ~0.55).

### pH modulates SspA channel activity

**Figure 5:**
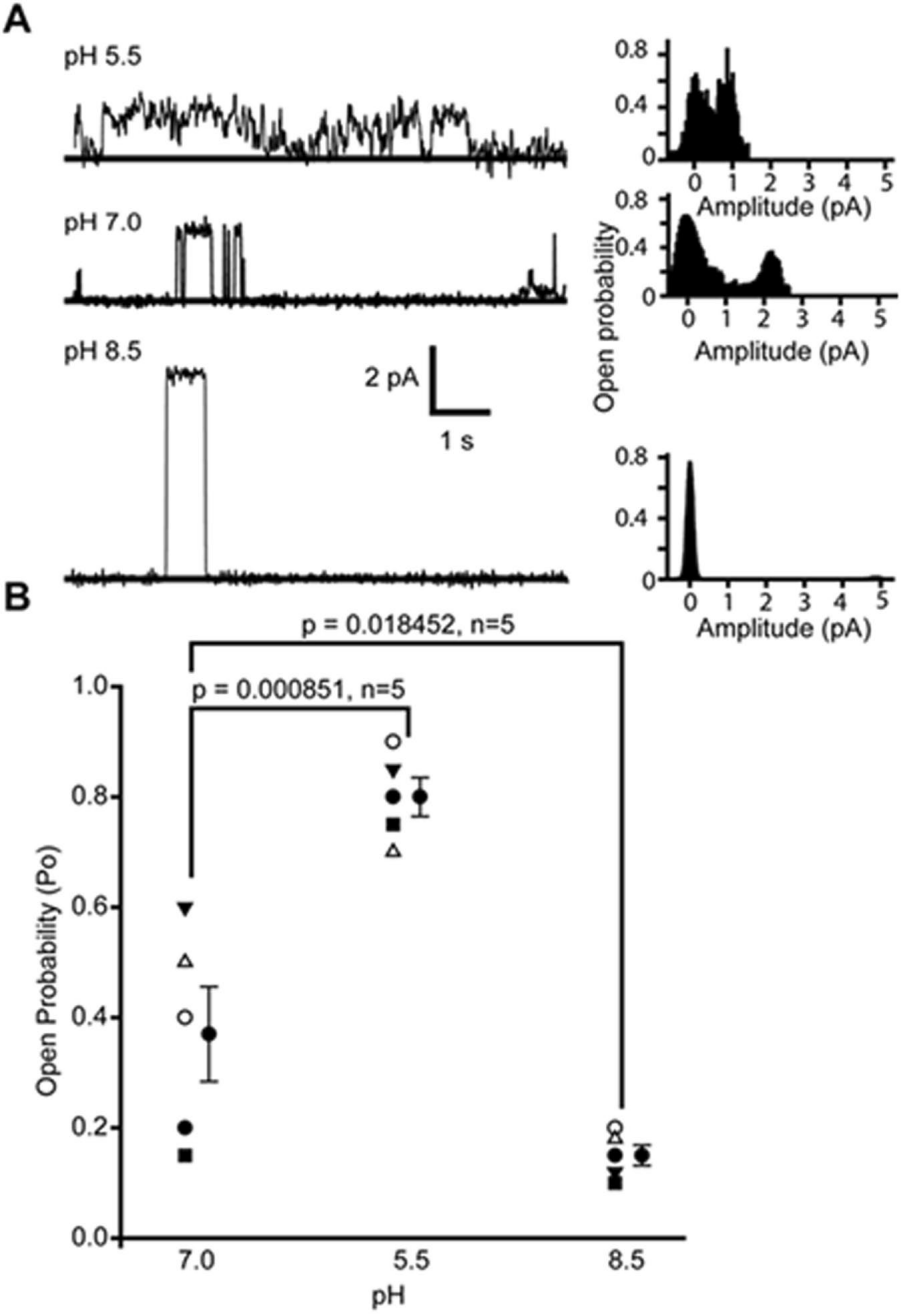
Regulation of SspA by pH. (**A**) Representative traces at pH 5.5, pH 7.0, and pH 8.5 along with their amplitude histograms. The solid line indicates the closed level. (**B**) Open probability of SspA-mediated currents at different pH conditions. The open probability increased significantly (p ≤ 0.001, n = 5) at pH 5.5 as compared to pH 7.0. At pH 8.5 the open probability significantly decreases (p ≤ 0.05, n = 5) as compared to pH 7.0.

Resistance to extreme acidic conditions allows bacteria to survive the low pH environment that they encounter in the gut of higher organisms. SspA expression is known to be essential for the survival of bacteria during acid-induced stress^19^. CLICs are known to show increased activity at lower pH^39, 40^; hence, we tested whether SspA-mediated currents are also sensitive to changes in pH. SspA proteins were incorporated into planar bilayers at pH 7.0, and pH was changed by adding either HCl or KOH on both sides (n = 5) of planar lipid bilayers. As shown in Fig. 5, consistent ion channel activity was obtained at pH 7.0 but on changing pH to 5.5, open probability of the channel significantly increased as reported for mammalian CLIC proteins^37, 38^. On further changing the pH to 8.5, the open probability was significantly decreased. (Fig. 5A and B). Even though the open probability increased at the lower pH, the current amplitude decreased and was similar to the sub-state level as observed for SspA (Figs 3, 4 and 5). At pH 8.5, sparse channel openings were observed, but surprisingly the current amplitude was ~4.8 ± 0.3 pA at + 100 mV. These results imply that the open probability as well as channel activity of SspA are directly modulated by pH, which should be further investigated in detail and in *in vivo* models.

### IAA-94 affects the growth of wild type but not the *sspA* mutant

**Figure 6:**
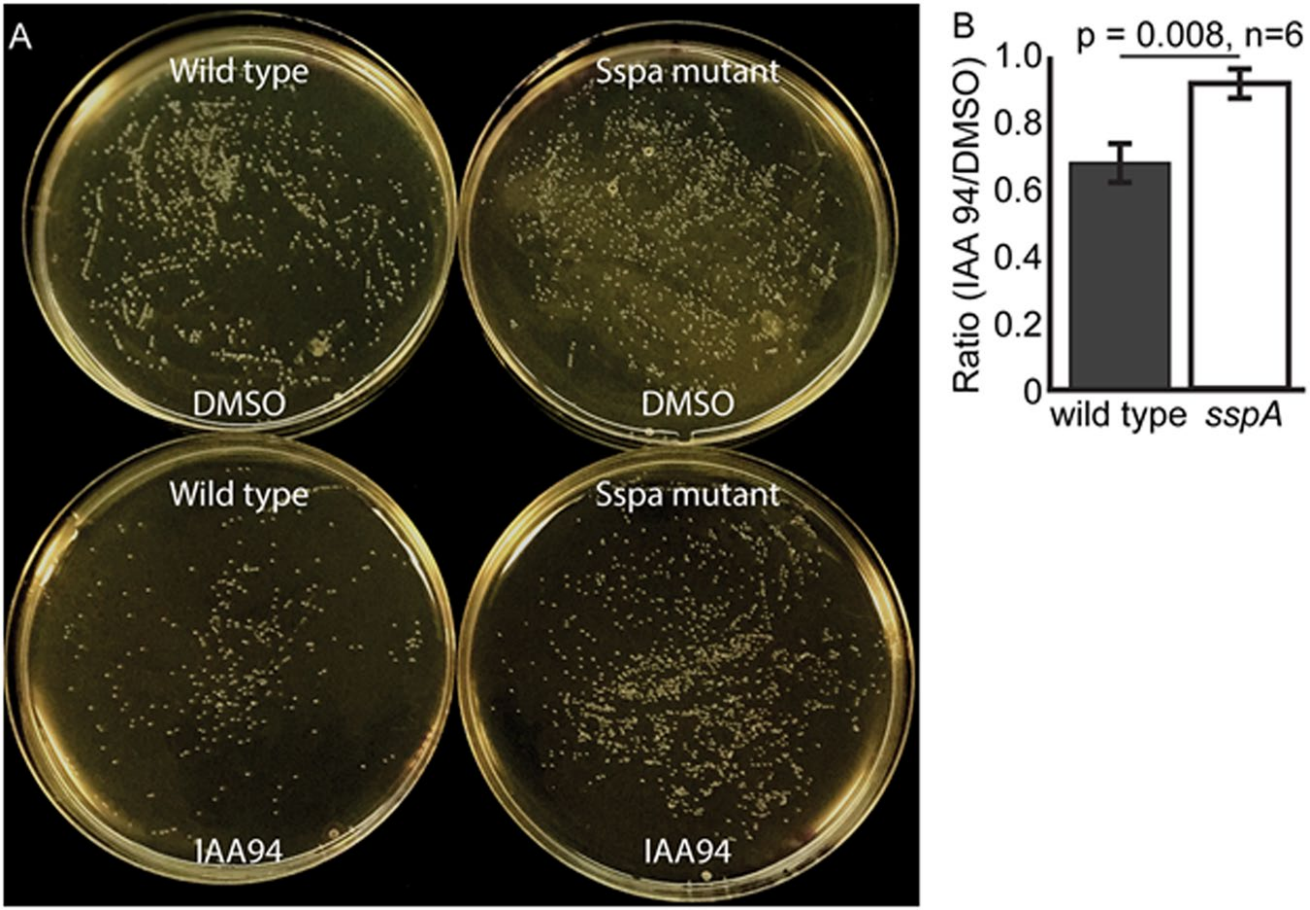
Effect of IAA-94 on *E. coli*. (**A**) *E. coli* growth on the LB agar medium with and without IAA-94. DMSO was used as a solvent control. Wild-type *E. coli* showed less number of colonies in the presence of IAA-94 but SspA mutant had no effect of IAA-94. (**B**) The ratio of a number of colonies present in IAA-94 vs DMSO for wild type and SspA mutant. IAA-94 significantly reduced the number of colonies (p ≤ 0.01, n = 6) for the wild type as compared to the SspA mutants.

Sensitivity to IAA-94 indicated the presence of a CLIC-like protein, SspA, in *E. coli* and, IAA-94 also blocked the activity of SspA in planar bilayers. Given IAA-94 showed a delay in the growth of *E. coli*, we tested the effect of IAA-94 on *sspA* null mutants. Null mutant and wild-type *E. coli* were cultured in presence or absence of 100 µM IAA-94 and the number of colonies was quantified (Fig. 6). The number of colonies in the wild-type strain was significantly reduced in the presence of IAA-94 but not in *sspA* null mutant strain (Fig. 6B). SspA mutants formed smaller colonies as compared to wild type. These results further confirm that SspA is a target of IAA-94, and is a CLIC homolog in prokaryotes.

## Discussion

Ion channels have evolved much before the appearance of complex mammals, but the functional studies of ion channels have long been dominated by mammalian models to understand the role of channels in cellular physiology. However, majority of the structure information of eukaryotic ion channels such as potassium and chloride channels is based on the crystal structure of bacterial channels^29, 41^. This shows the evolutionary relatedness of organisms and how prokaryotic ion channels can contribute to the understanding of general principles underlying ion channel structure and functional relationship.

Chloride (Cl) channels were first studied in *Torpedo electroplax* ^42^ but they are known to have several prokaryotic homologs^43^. Studies in bacteria revealed unique physiological roles of Cl channels. For example, Cl channels from *E. coli* (ClC-ec1) resemble exchangers rather than channels^44^. CLIC proteins were first purified from mammalian kidney and trachea by affinity purification using IAA-94^2^ and the functional roles are studied only in eukaryotes. Characterization of prokaryotic homologs of CLICs such as SspA can provide further insights to understand the structure-function relationship as well as physiological roles of these proteins.

CLICs are a major group of ion channels present in the intracellular organelles^45–47^ and are not found at the plasma membrane^1^. So far, the electrophysiological evidence for channel formation has been shown only for eukaryotic CLIC proteins. Our sequence analysis shows that CLIC-like proteins exist in prokaryotes and are known as SspA in Gram-negative bacteria. They cluster along with omega GSTs. By sequence homology, we have found several bacterial homologs of CLIC proteins with conserved secondary structure elements. Similar to CLICs, there are two hydrophobic domains present in the N- and C-termini regions, respectively. In addition, SspA has several conserved leucine residues, which could play a role in the conservation of higher-order structures. These similarities indicate that higher organism CLICs must have been evolved from their bacterial counterparts. However, it needs to be investigated if channel activities exist in other classes of prokaryotic organisms, such as archaea to build the complete trail of CLIC proteins in evolution.

Mammalian CLICs exist as soluble and membrane forms, and the soluble forms are known to be localized in the cytoplasm as well as a nucleus. CLIC4 translocates to the nucleus under stressed conditions and consists of a nuclear localization signal^48^. The nuclear localization of CLICs is thought to be associated with transcription of genes. CLIC4 is shown to interact with BMP pathway transcription factor Schnurri-2 and with Smad proteins, thus plays a role in modulating the activity of TGF-β signaling^48^. Interestingly, SspA is associated with RNA polymerase^24^ and is required for the transcriptional activation of phage P1 late genes^25^. SspA is also involved in regulating stationary phase induced stress response by decreasing the levels of H-NS, a global regulator of acid tolerance. SspA actively contributes to the expression of virulence genes in several bacteria^19, 20, 49, 50^. These studies imply that CLICs have possibly retained the transcriptional role of SspA although the actual mechanism and the genes they affect are still unclear.

The secondary and tertiary structure comparison of CLIC proteins to bacterial SspAs indicate that they share significant similarities. The crystal structure of *Yp* SspA shows similar structural homology to GSTs indicating their close relation of these proteins to the omega class of GSTs as reported earlier^11, 18^. *Yp* SspA also shares substantial tertiary structural similarity to several mammalian, *Ce* and *At* CLICs that we compared, further reinforcing our hypothesis that CLIC proteins share their roots with bacterial SspA proteins.

*Ec* SspAs are structurally similar to *Yp* SspA and form ion channels similar to CLICs. Single-channel conductance and sub-state level conductance of *Ec* SspA were analogous to the single-channel conductance of mammalian CLIC4^33, 34^. Further, *Ec* SspAs failed to form ion channels under strongly oxidizing conditions possibly due to oligomerization and inability to insert into planar bilayers. CLIC proteins are shown to oligomerize under oxidizing conditions and cysteine residues are known to play a key role in their trans-redox regulation^32, 34^. It is not clear whether these bacterial channels could be regulated by redox potential since we did not observe activity in strong oxidizing conditions. In addition, lack of an N-terminus cysteine in *Ec* SspA will rule out any trans-redox regulation similar to mammalian CLICs^32^. The only cysteine residue present in *Ec* SspA is located in the C-terminus region.

IAA-94 was employed by Landry *et al*., to purify CLIC-like proteins from kidney and trachea^2^, and an open probability of CLIC1 proteins decreased in the presence of IAA-94^32, 38^. Blockage of *Ec* SspA-mediated currents by IAA-94 provided a direct pharmacological evidence that *Ec* SspA-mediated currents are similar to CLIC proteins, and not to other chloride channels such as CFTR^51^ and ClCs^52^. In addition to blocking the ion channel activity^37, 53^, IAA-94 also blocked the glutaredoxin-like activity of CLIC1 proteins^54^. Although SspA assumes the GST fold, it is shown to lack enzymatic activity that requires GSH as a cofactor^23^. Surprisingly, IAA-94 delayed the growth but not the viability of *E. coli*. As SspA is highly expressed under stress^19^, the addition of IAA-94 could have caused stress-induced overexpression of SspA in *E. coli*. The overexpression of SspA will result in an increase in survivability of *E. coli*, which possibly allowed the IAA-94 treated bacteria to reach the stationary phase.

Even though CLICs are named as chloride channels^55^; they are known to form non-selective ion channels^1, 32–34^ in planar bilayers. We have found that *Ec* SspA also forms non-selective, perhaps, cation-selective ion channels in KCl gradient (Fig. 3). Only in the presence of Tris, a larger cation, CLIC proteins are known to form highly chloride selective channels^32^, and *Ec* SspA also forms highly chloride-selective channels in the presence of Tris. This could be attributed to the presence of a larger cation, which is not permeable through *Ec* SspA channels. However, the decrease in single-channel conductance indicates that K^+^ ions have significant contribution in the channel conductance. In the presence of K^+^ ions, the channel can allow both K^+^ and Cl^−^ but is more selective for K^+^, however, when K^+^ is not present, the channel can still allow Cl^−^ albeit with lower conductance. In either case, the selectivity of *Ec* SspA channels, much like CLIC proteins, is poor for anions in the presence of a smaller cation K^+^ in planar bilayers. Non-selective nature of *Ec* SspA can indicate evolutionary significance and adaptation for salt tolerance that increases only under specific conditions.

Although planar bilayers provide a highly sensitive technique for studying ion channels, the reconstitution method could affect the protein conformations, which in turn can alter the biophysical properties such as single channel conductance, regulation by accessory subunits and selectivity of the ion channel. The current study on SspA and previous studies on CLICs in planar bilayers need to be corroborated in native environments to determine their ionic selectivities. It is possible that CLICs are more chloride selective in physiological conditions given it is known to play a significant role in organelle and cellular acidification^53, 56^.

The pore-forming region of mammalian CLICs is located in the N-terminus^1^. However, in the absence of crystal structure of membrane forms, the defined arrangement of amino acids in the pore region is not deciphered. Earlier the C24 in CLIC1 was shown to be present just outside the pore region where it is involved in trans-redox regulation of ion channel activity^32^. In the proposed model, arginine 29 (R29) and lysine 37 (K37) of CLIC1 were predicted to face the pore region^1^. These residues were recently shown to be involved in modulating the biophysical properties of the CLIC1 channel supporting the earlier model that R29 and K37 form two rings at the top and center of the channel^57^. The CLIC1 K37A mutation increased the single channel conductance without altering the selectivity and CLIC1 R29A affects the voltage sensitivity of the channel. However, R29 and K37 are not conserved in all the CLICs across different species. CLIC1 R29 is replaced by glutamine (Q) in SspA and CLIC1 K37 is substituted by glycine (G) in *C. elegans* (Figs 2 and 4). The residue which was conserved in the transmembrane domain across the mammalian CLIC homologs and CLICs in other species is leucine 29 (L29) of *Ec* SspA (CLIC1 L34). On mutating SspA L29A, a significant increase in single channel amplitude and conductance was observed while the channel remained non-selective. The open probability of the channel was not altered by L29A substitution and was similar to the wild-type. Leucine is known to interact with the hydrophobic lipid bilayer^58^ and substituting it with alanine potentially alters this interaction. This disruption in lipid interaction could have possibly altered the stability of the pore region as also seen in hyperpolarization-activated cyclic nucleotide-gated channels^58^. SspA is more selective for K^+^ but SspA L29A was non-selective under similar conditions. Association of a substate with the larger channel opening also indicates a possible transition from smaller pore to wider pore. However, further structure-function analysis will provide information on all the residues lining the hydrophilic and hydrophobic regions of the pore.

SspA is proposed to be involved in cell-cell communications under stress conditions. *E. coli* lacking SspA is less viable than wild type during stresses such as prolonged starvation^26^ and stationary phase-induced acid tolerance^19^. On these lines, CLICs share similar behavior and response to different stress conditions^59^ and even translocate to various cellular compartments^60^ indicating a functional relation between two proteins. SspA is highly conserved in Gram-negative bacteria^23^. It is hypothesized that mitochondria are formed from a symbiotic association of bacteria (specifically Gram-negative)-like organisms with the eukaryotic cells^61^. It makes perfect sense that intracellular ion channel proteins are only found in organelles inside the cells and might suggest their evolution along with the integration of bacteria into eukaryotic cells forming mitochondria and other organelles^62^. Recently, CLIC4 and CLIC5 were shown to be localized to the mitochondrial membranes^45, 46^. It is also interesting that SspAs maintain acid balance as well as tolerance^23^, and CLICs are known to perform similar functions in eukaryotic cells^39, 63^. *E. coli* has to survive in the extremely acidic environment and SspA is essential for its survival as shown earlier^23^. In our study, we discovered that activity of SspA increases in lower pH and channels were relatively silent in a basic environment. These results could explain how *E. coli* can survive the lower pH in the gut and SspA-mediated channels play an important role in this process.

Taken together‚ our study shows that CLIC proteins are evolutionarily significant, functionally conserved and that they could have evolved from bacteria. Investigation of functional roles of SspAs could provide vital information on physiological implications of CLIC proteins in mammals.

## Experimental Procedures

### *E. coli* growth analysis

*E. coli* (DH5α) cultures were grown in the Luria-Bertani (LB) medium at 37 °C on an orbital shaker. DMSO and IAA-94 (1, 30 and 100 µM) were added to the medium. Optical density (O.D.) was measured every 60 min. Growth curves for O.D. vs. time were plotted and 50% growth was measured from fitted curves on the SigmaPlot 12.5 (Systat Software, Inc. San Jose, CA).

### *In silico* analysis

CLIC sequences (*Hs* CLIC1: NP_001274523.1, *Hs* CLIC2: NP_001280.3, *Hs* CLIC3: NP_004660.2, *Hs* CLIC4: NP_039234.1, *Hs* CLIC5a: NP_001107558.1, *Hs* CLIC5b: NP_058625.2, *Hs* CLIC6: Q96NY7.3, *Dm* CLICa: NP_572928.1, *Dm* CLICb: NP_001259537.1, *Dm* CLICc: AGB95379.2, *Ce* EXC4: AAQ75554.1, *Ce* Exl1: NP_507142.2, *At* DHAR1: Q9FWR4.1, *At* DHAR2: Q9FRL8.1, *At* DHAR3: Q8LE52.1, *At* DHAR4: Q9FG59.1) were searched for homologs against prokaryotic databases using the BLAST algorithm^31^ and homologs were found (*Yp* SspA: AAS63962.1, *Nm* SspA: NP_274947.1, *Ec* SspA: EGT68941.1). All the sequences were aligned using CLUSTAL W omega and a phylogenetic tree was built using CORAL. For *Ce* GST, the NP_507142.2 sequence was used. The crystal structures of CLICs, *Torpedo electroplax* ClC and *Yp* SspA were analyzed using PyMOL and compared using SSM (PDB eFold)^64^, DALI^65^ and CE^66^ servers.

### Construction of L29A mutants

The site-directed mutant was generated by quick change method (Stratagene) followed by *DpnI* digestion. Briefly, sense (5′ GTC GCC ATT GTG GCA GCT GAG AAA GGT 3′) and antisense (5′ ACC TTT CTC AGC TGC CAC AAT GCG GAC 3′) primers were used to generate the mutant using full-length plasmid as a template DNA. The PCR conditions were 95 °C for 2 min, 18 cycles of 95 °C for 30 s, 58 °C for 30 s and 72 °C for 5 min followed by extension for 10 min at 72 °C. The reaction mixture was digested with *DpnI* enzyme (1 hour) followed by transformation of *E. coli* (DH5α) cells. Clones were screened by digesting the mutant plasmid with *PvuII* restriction enzyme which was introduced in primers and further confirmed by sequencing.

### Expression and purification of SspA

Recombinant *Ec* SspA protein was expressed and purified as described earlier^25^. Briefly, SspA was expressed from DJ624 (MG1655 *lacX74 mal*::*lacIq*) containing pDJ706, encoding SspA with hexahistidine residues at the N-terminus. The bacterial culture containing pDJ706 was grown at 30 °C in the Luria-Bertani medium in the presence of 100 µg mL^−1^ ampicillin. The culture was allowed to grow to an OD_600_ of ~1.0 without induction and harvested by centrifugation. The cells were resuspended in buffer A [20 mM Tris-HCl, 100 mM NaCl at pH 8.0 and 0.1 mM phenylmethylsulphonyl fluoride (PMSF)]. Resuspended cells were ruptured using a French press at a maximum pressure of 900 p.s.i. To remove unbroken cell debris and insoluble fractions, cell lysate was centrifuged at 4 °C for 30 min at 19,000 g. The supernatant was collected and overexpressed proteins were purified from the supernatant using Talon metal affinity resin (BD Biosciences) according to the manufacturer’s instructions. Bound proteins were eluted in buffer B (Buffer A supplemented with 100 mM imidazole) and dialyzed overnight against TGED buffer [10 mM Tris-HCl, 300 mM NaCl, 0.01 mM EDTA, 0.1 mM dithiothreitol (DTT), 5% (*v/v*) glycerol at pH 8.0]. Dialyzed and the equilibrated solution was passed over a MonoQ column equilibrated with TGED buffer and eluted with a gradient NaCl concentration (0.2 M−0.5 M NaCl in TGED). The peak fractions of SspA, eluted around a NaCl concentration of 0.35 M, were collected and concentrated using Microcon-10 concentrators (cut-off value of 10,000 Da; Amicon) according to the supplier’s manual. Purified proteins were stored at −80 °C until further use. Similar expression and purification protocol were followed for the L29A mutant.

### Reconstitution in planar lipid bilayers

Lipid bilayers were prepared at room temperature from POPE, POPS, and cholesterol (Avanti Polar Lipids, Alabaster, AL) in the molar ratio of 4:1:1 as described earlier^32^. Lipids were dried with nitrogen gas and resuspended in n-decane (25 µg total lipid/µL). Lipid solution was applied across a 0.2 mm orifice in a polystyrene cuvette. The cuvette was fitted into a black Delrin chamber partition; across the cuvette was designated *cis* and *trans*, and filled with recording solutions. The *cis* chamber was voltage-clamped by a bilayer clamp amplifier (BC-535, Warner Instruments) using agar salt bridges, and an 8 pole low-pass Bessel filter (Warner Instruments). Lipid bilayers formed had a typical capacitance of 105 ± 10 pF (n = 30). Bilayers were formed in 500 mM KCl *cis vs*. 50 mM KCl *trans* (all the solutions contained 10 mM Tris-HCl, pH 7.4 and 1 mM DTT unless otherwise specified), and up to 25 ng/mL recombinant, SspA proteins were added into the *cis* chamber. A solution containing proteins were stirred and currents normally appeared within 10 minutes. Currents are labeled as positive or negative following the standard convention (i.e., positive currents represent net cation flux from *cis* to *trans*).

Single-channel currents were recorded using pClamp10.1 (Axon Instruments) and analyzed using current *vs*. voltage (I/V) plots. The data were filtered at 1.0 kHz. For selectivity, reversal potential obtained from I/V plots was used in the following form of Goldman-Hodgkin-Katz voltage equation;

1 $$\frac{Panion}{Pcation}=\frac{\{n.\,\exp (\frac{Er}{k})-1\}}{\{n-\exp (\frac{Er}{k})\}}$$

where n is the cis/trans salt activity ratio, E_r_ is the reversal or equilibrium potential and

2 $$k=\frac{{\rm{RT}}}{{\rm{F}}(26\,{\rm{mV}})}$$

R is the universal gas constant (8.314 JK^−1^mol^−1^), T is the absolute temperature, F is the Faradays constant (9.6485 × 10^4^ Cmol^−1^).

For inter anionic selectivities, salt concentrations were corrected and relative anion permeabilities (*P*) were calculated from the Nernst equation adapted for bi-ionic conditions;

3 $$\frac{Panion}{PCl}=\frac{a[Cl]cis}{a[anion]trans}\,\times \,\exp (-\frac{zFEr}{RT})$$

where a is the activity coefficient of the relative salt, z, F, R and T have their standard significance.

Channels were also reconstituted in the presence of 500 mM TrisCl *cis* vs. 50 mM TrisCl *trans* (pH 7.4 and 1 mM DTT) and E_r_ was calculated from the I/V curve in Tris gradient solutions. To modify pH, HCl or KOH were added after a channel was successfully incorporated and recorded.

### Wild-type and SspA mutant bacterial strain growth

Fresh overnight cultures of MG1655 (wild type) and its *sspA* mutant derivative^19^ were diluted 1/100 and grown till the absorbance reaches 0.4 OD. The cultures were further diluted to 1/5000 and 20 μl of the same was plated on LB agar plate containing kanamycin (50 μg/ml) in the presence of DMSO (vehicle control) or 100 μg/ml IAA-94. Plates were incubated at 30 °C. A total number of colonies was quantified using Image J software and the ratio of a number of colonies in the presence of IAA-94 and DMSO were calculated.

## Electronic supplementary material

SUPPLEMENTARY TABLE 1

Supplementary Figure 3F
