# Supplementary material for: Identification and Characterization of a Bacterial Homolog of Chloride Intracellular Channel (CLIC) Protein
Source: Sci Rep. 2017 Aug 17;7:8500. doi: 10.1038/s41598-017-08742-z (PMC5561075; doi:10.1038/s41598-017-08742-z)
Supplement: Supplementary file 1 — SUPPLEMENTARY TABLE 1 [file 41598_2017_8742_MOESM1_ESM.doc]

**Identification and Characterization of a Bacterial Homolog of Chloride Intracellular Channel (CLIC) Protein Family**

Shubha Gururaja Rao^1^, Devasena Ponnalagu^1^, Sowmya Sukur^1^, Harkewal Singh^2^, Shridhar Sanghvi^1^, Yixiao Mei^1^, Ding J. Jin^3^, and Harpreet Singh^1,4*^

**SUPPLEMENTARY TABLE 1. Sequence alignment and identity score for various CLICs, GST and SspA proteins.**
