# Supplementary material for: Identification and Characterization of a Bacterial Homolog of Chloride Intracellular Channel (CLIC) Protein
Source: Sci Rep. 2017 Aug 17;7:8500. doi: 10.1038/s41598-017-08742-z (PMC5561075; doi:10.1038/s41598-017-08742-z)
Supplement: Supplementary file 2 — Supplementary Figure 3F [file 41598_2017_8742_MOESM2_ESM.doc]

**Identification and Characterization of a Bacterial Homolog of Chloride Intracellular Channel (CLIC) Protein Family**

Shubha Gururaja Rao^1^, Devasena Ponnalagu^1^, Sowmya Sukur^1^, Harkewal Singh^2^, Shridhar Sanghvi^1^, Yixiao Mei^1^, Ding J. Jin^3^, and Harpreet Singh^1,4*^

**SUPPLEMENTARY FIGURE 3 F Uncropped polyacrylamide gel for Fig 3 F.**


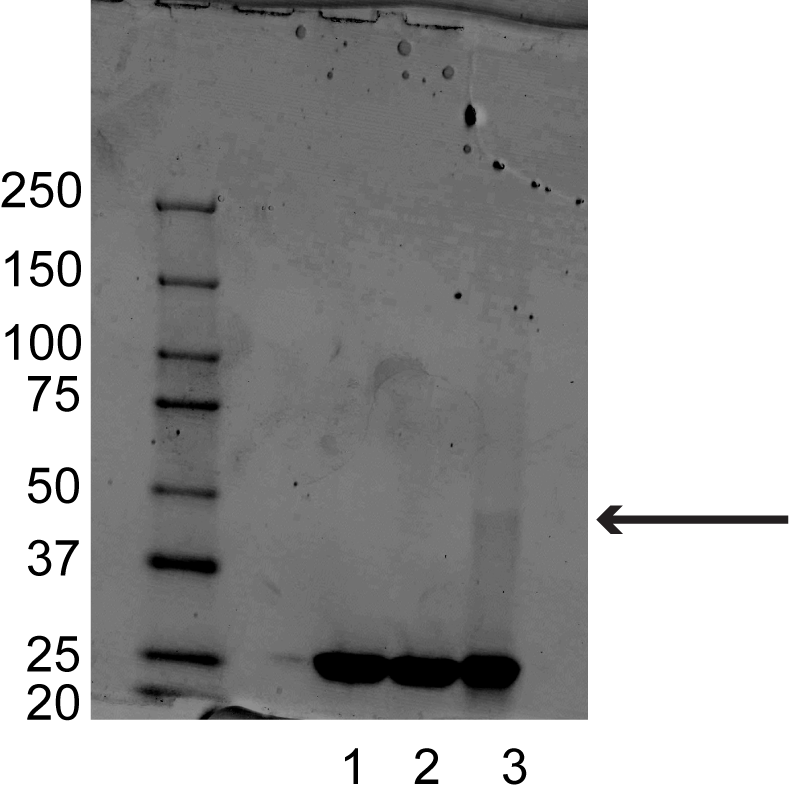


**Supplementary Figure 3F**. Polyacrylamide gel stained with Coomassie brilliant blue showing SspA oligomerisation in H_2_O_2_ (lane 3, arrow) but not in the presence of 1 mM and 5 mM DTT (lane 1 and lane 2, respectively).
